# Poor condition and infection: a vicious circle in natural populations

**DOI:** 10.1098/rspb.2008.0147

**Published:** 2008-04-30

**Authors:** Pablo M Beldomenico, Sandra Telfer, Stephanie Gebert, Lukasz Lukomski, Malcolm Bennett, Michael Begon

**Affiliations:** 1 School of Biological Sciences, University of LiverpoolCrown Street, Liverpool L69 7ZB, UK; 2 Facultad de Ciencias Veterinarias, Universidad Nacional del LitoralRP Kreder 2805 3080 Esperanza, Santa Fe, Argentina; 3 National Centre for Zoonosis Research, University of LiverpoolChester High Road, Neston CH64 7TE, UK; 4 Global Health Programs, Wildlife Conservation SocietyBronx, NY 10460, USA

**Keywords:** disease ecology, population dynamics, wildlife health, host–parasite interaction

## Abstract

Pathogens may be important for host population dynamics, as they can be a proximate cause of morbidity and mortality. Infection dynamics, in turn, may be dependent on the underlying condition of hosts. There is a clear potential for synergy between infection and condition: poor condition predisposes to host infections, which further reduce condition and so on. To provide empirical data that support this notion, we measured haematological indicators of infection (neutrophils and monocytes) and condition (red blood cells (RBCs) and lymphocytes) in field voles from three populations sampled monthly for 2 years. Mixed-effect models were developed to evaluate two hypotheses, (i) that individuals with low lymphocyte and/or RBC levels are more prone to show elevated haematological indicators of infection when re-sampled four weeks later, and (ii) that a decline in indicators of condition is likely to follow the development of monocytosis or neutrophilia. We found that individuals with low RBC and lymphocyte counts had increased probabilities of developing monocytosis and higher increments in neutrophils, and that high indices of infection (neutrophilia and monocytosis) were generally followed by a declining tendency in the indicators of condition (RBCs and lymphocytes). The vicious circle that these results describe suggests that while pathogens overall may be more important in wildlife dynamics than has previously been appreciated, specific pathogens are likely to play their part as elements of an interactive web rather than independent entities.

## 1. Introduction

Pathogens are detrimental to their hosts. By extracting the host's resources and/or inducing a nutritionally demanding immune response, even relatively benign endemic pathogens seem likely to exert a negative effect on reproduction and/or survival, as has recently been shown for cowpox virus in field voles (*Microtus agrestis* L.: Burthe *et al*. 2008). On the other hand, the dynamics of infection might depend on the hosts' vulnerability, as poor condition is likely to predispose individuals to infectious and parasitic diseases (Nelson & Demas 1996; Tompkins & Begon 1999). Variation in condition is itself likely to be related to host dynamics and abundance: condition is poorest when host abundance is high and competition for resources is intense (Begon *et al*. 2006). Pathogen prevalence will also often be the highest at higher host abundances (Hudson *et al*. 2002), but those pathogens may in turn reduce host abundance. There is a clear potential for synergy between infection and condition: poor condition predisposes to host infections, which further reduce condition and so on. Between them, they may exert far greater adverse effects on hosts at higher abundances than either would do alone, and hence may influence host dynamics in ways neither could do alone. There is a need for empirical data to support this notion.

To date, field studies relating condition to infection in natural populations are scarce, and most of them are cross-sectional surveys that fail to address the temporal relationship between cause and effect (e.g. Hakkarainen *et al*. 2007). Therefore, an association between infection and poor condition might mean that the disease caused the poor condition or that the individual became infected owing to its poor condition, or both.

Haematological parameters can be used to assess condition and infection in wild populations (Beldomenico *et al*. in press). Poor condition in an individual is usually reflected by low peripheral lymphocyte counts (lymphopoenia, an indication of immunosuppression or poor immunological investment) or low red blood cell (RBC) counts (anaemia, an indication of poor aerobic capacity), whereas some infections can be detected by elevated neutrophil counts (neutrophilia, an indication of acute inflammatory response) or high monocyte counts (monocytosis, a reflection of chronic inflammatory response).

Here, we describe a longitudinal study in replicated wild field vole populations, which attempts to evaluate the temporal sequence of the relationship between condition and infection by testing two hypotheses, (i) that individuals with lymphopoenia and/or anaemia are more prone to show elevated haematological indicators of infection when re-sampled four weeks later and (ii) that a decline in indicators of condition is likely to follow the development of monocytosis or neutrophilia.

## 2. Material and methods

### (a) Sampling procedures

In Kielder Forest (Northumberland, UK), three sites with suitable habitat for field voles were sampled (‘primary sessions’) every four weeks over a 2-year period (from April 2005 to March 2007) apart from eight-week gaps between November and February (Beldomenico *et al*. in press). At each site, a trapping grid measuring 50×50 m was established, with 100 Ugglan special live capture traps (Grahnab, Sweden), set at approximately 5 m intervals. In each primary session, the traps were checked for capture five times, at sunrise and before sunset (an approx. 12 hour interval).

Individuals were uniquely and permanently marked on first capture with a small microchip transponder (Labtrac by AVID plc, UK). On first capture within a primary session, each vole was assessed for pelage (juvenile coat, first moult, adult coat), sex and body mass (to the nearest 0.5 g, using a spring balance).

### (b) Haematological parameters

As described in detail by Beldomenico *et al*. (in press), a haemogram was produced with blood collected from the tip of the tail of live individuals. Briefly, 2 μl of non-coagulated blood were diluted 1 : 20 in 4% acetic acid with 1% crystal violet and 1 : 5000 in PBS, to count WBCs and RBCs, respectively, using Kova Glasstic slides with grids and hence to determine their concentration. The rest of the blood sample was used to produce blood smears for differential WBC counts, which allowed the proportion of each WBC type and their concentration to be estimated. Smears were air dried, fixed in methanol and stained with Rapid Romanowsky Stain Pack—HS705 (HD Supplies, Aylesbury).

RBCs (millions per μl) and lymphocytes (cells per μl) were the proxies of condition. For indicators of infection, we used levels of neutrophils and monocytes. The response to infection was approximated by assessing the presence of neutrophilia or monocytosis. Individuals with more than 2000 neutrophils μl^−1^ were considered to be displaying neutrophilia, as this was the value below which approximately 80% of measurements lay during the non-breeding period and was the median for spring, when neutrophil counts peak (Beldomenico *et al*. in press). The presence of chronic infection was indicated by monocytosis. The same criterion used for neutrophils determined that the cut-off value for monocytosis was 1500 μl^−1^.

### (c) Statistical analysis

The analysis was restricted to data collected from April to November 2005 and from March to November 2006, as the longer trapping intervals during winter precluded the assessment of monthly variation in condition at the individual level. Nevertheless, infection rates as indicated by monocytes and neutrophils were at their lowest levels in winter (Beldomenico *et al*. in press). The study unit was a vole month: that is, an individual vole followed from one primary trapping session to the next, four weeks later. Juveniles were omitted from the analysis, as changes in RBC and lymphocyte counts during the first weeks of life are drastic (Beldomenico *et al*. in press).

In general, analyses were conducted using linear mixed-effect models (LMMs) with random intercepts or generalized linear mixed models (GLMMs), using the statistical software package R (The R Foundation for Statistical Software). In model selection, terms were eliminated from a maximum model (Beldomenico *et al*. in press) to achieve a simpler model that retained only the significant main effects and interactions, using the Akaike information criterion (AIC; Akaike 1974). Terms were eliminated if they did not reduce the AIC by more than two units when included.

The unique identification number given to each vole (VOLE_ID) was used as a random effect to account for correlation among observations of the same individual. As voles sampled at the same site in the same month shared the same population-level covariates, analyses also included the interaction between site and month as a random effect (SITE×SESSION; Telfer *et al*. 2005). However, due to the relatively low number of captures per animal (mean=2.2), there were problems including VOLE_ID as a random effect in the maximum model. VOLE_ID was therefore added to the restricted model to verify that accounting for non-independence of observations from the same animal did not alter the results.

Besides the variables of interest, the analysis considered the following fixed effects. The sample year was indicated by the categorical variable YEAR (a description of population densities for all sites during the study years, as well as the preceding dynamics, is reported by Beldomenico *et al*. in press) and males and females distinguished by the variable SEX. Age was approximated using weight (WGT; Weight+Weight^2^ when appropriate). The variable R/N indicated whether individuals were recaptured (‘R’) or newly captured (‘N’). Seasonality was best reflected in the maximum models (other analyses not shown) by using two sinusoidal components (SEASON[sin]+SEASON[cos]) (Beldomenico *et al*. in press). Population sizes were also included: both current estimates (DENSITY-0) and densities at different lags (the lags evaluated were those found important for a particular cell level by Beldomenico *et al*. in press). These were estimated using Huggins's closed capture models within a robust design (Huggins 1989; Kendall & Nichols 1997). The analysis was conducted in program Mark using mixture models (Pledger 2000) to allow heterogeneity in capture probabilities.

### (d) Hypothesis 1: does poor condition precede infection?

Two analyses were conducted, each with a different haematological indicator of infection as the response variable.

The subsets of data used depended on the response variable. When the response explored was monocytosis in the second sample (yes/no), only observations of individuals without monocytosis in the first sample (four weeks earlier) entered the analysis (*n*=771). Because a four-week gap may be too long to evaluate the development of an ephemeral response such as neutrophilia, we explored instead the difference between neutrophil levels in the first and second samples (i.e. change in neutrophil count in a month; *n*=1047).

The analyses were conducted using a GLMM with a binary response (monocytosis) and an LMM for neutrophil-level variation (as the distribution did not substantially depart from normality). The explanatory variables evaluated (plus their pairwise interactions) were SEASON, DENSITY, WGT, R/N, SEX, YEAR, RBCs and Lymphocytes. Different DENSITY lags were assessed by AIC in the maximum models. When examining the linearity of the logit in the GLMM for continuous explanatory variables, it was noted that ‘Lymphocytes’ needed to be polynomial.

The focus of the results and discussion is the associations between indicators of infection and indicators of condition. Other variables were included to control for confounding and interaction phenomena (Kleinbaum *et al*. 1998).

### (e) Hypothesis 2: is infection followed by poorer condition?

Two analyses were conducted, one with change in RBC counts between successive samples (millions per μl; *n*=923) as the response, and the other with change in lymphocyte counts (lymphocytes per μl; *n*=970). The explanatory variables included in the maximum model were SEASON, DENSITY, WGT, R/N, SEX, YEAR, but the variables of focal interest were current presence (i.e. at sample 1) of monocytosis and neutrophilia.

## 3. Results

### (a) Condition preceding elevation in indicators of infection

**Figure 1:**
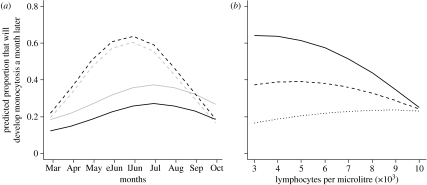
(*a*) Predicted development of monocytosis by sex (grey, female; black, male). Simulation for recaptured (‘R’) 22 g individuals at fixed past density (50 voles per grid) in 2005. In the simulation, ‘anaemic’ (dashed lines) are individuals with 3 million RBCs μl^−1^, and ‘normal’ (solid lines) are voles with 8 million RBCs μl^−1^ (eJun, early June; lJun, late June). (*b*) Predicted development of monocytosis for different levels of lymphocytes and population densities (solid line, 30 voles per grid; dashed line, 60; dotted line, 90). Simulation for 22 g recaptured (‘R’) males in June of 2005.

The final model assessing the variables preceding monocytosis is detailed in table 1. During 2005, the probability of developing monocytosis during spring (the peak season for monocytes) was much greater for anaemic individuals than for normocytic ones and the peak occurred earlier, particularly for males (figure 1*a*). In spring 2006, however, RBC levels did not appear to be associated with the development of monocytosis in males, while anaemic females were *less* likely to develop monocytosis. (Adult females developing monocytosis after March 2006 had a mean RBC count greater than 9 millions μl^−1^ compared with less than 4 millions μl^−1^ for those not developing monocytosis; *t*-test *p*<0.001.)

The effect of lymphocyte levels on monocytosis varied with host density. For individuals with high lymphocyte levels, the probability of developing monocytosis was relatively low at all densities. But whereas the probability of developing monocytosis increased as lymphocyte levels declined at low densities (figure 1*b*), at higher densities the probability of developing monocytosis was lower still among lymphopoenic individuals.

**Figure 2:**
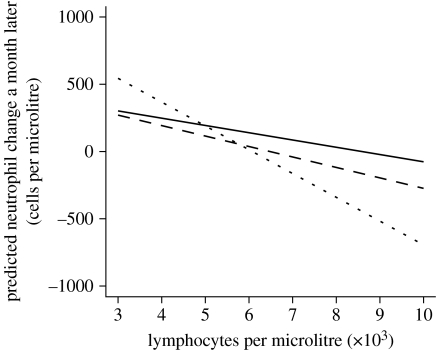
Predicted neutrophil change by weight (dotted line, 35 g; solid line, 25 g; dashed line, 17 g). Simulation for recaptured (‘R’) males in April at a fixed past density (50 voles per grid).

The final model assessing the variables associated with the fluctuations in circulating neutrophils is detailed in table 2. Independent of any other factor evaluated, anaemic individuals tended to exhibit an increase in neutrophil counts while normocytic ones tended to exhibit a decline. Neutrophil levels also increased among lymphopoenic individuals but decreased on average for voles with higher lymphocyte levels, particularly heavier individuals (figure 2).

### (b) Change in RBC and lymphocyte counts following neutrophilia or monocytosis

**Figure 3:**
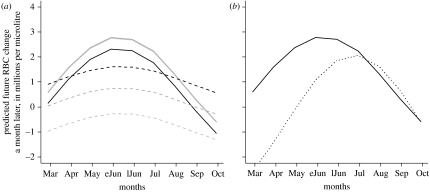
Simulations of the LMM describing changes of RBC counts in four weeks following (*a*) monocytosis (dashed, monocytosis; solid, normal) or (*b*) neutrophilia (dotted line, neutrophilia; solid line, normal). Three different weights (black, 17 g; dark grey, 22 g; light grey, 35 g) were simulated in (*a*), but in (*b*) the simulation corresponds to a 22 g vole. Current host density was fixed at 90 voles per grid, the average population size in summer–autumn (eJun, early June; lJun, late June).

Table 3 shows the final models describing the effects of monocytosis and neutrophilia on subsequent changes in lymphocyte and RBC counts. Individuals with monocytosis showed a substantial decline in lymphocyte counts (approx. 2000 lymphocytes μl^−1^ fewer, four weeks later), whereas on average, individuals without monocytosis tended not to decline. A negative impact of monocytosis on RBCs (figure 3*a*) was particularly evident when current densities exceeded 70 individuals per grid (population estimates ranged from 10 to 80 in spring, and from 50 to 140 in summer–autumn) and in heavy individuals. However, among light individuals, individuals *without* monocytosis were more likely to show declines in RBC levels, particularly in spring.

Neutrophilia was associated with negative changes of lymphocyte counts only in 2006, and it was followed by deeper declines of RBC counts in spring, particularly at high densities (figure 3*b*).

## 4. Discussion

While in human medicine, evidence of malnutrition and immunosuppression as important risk factors for infectious disease is overwhelming (e.g. Piyathilake *et al*. 2004), the current evidence available in wildlife consists only of a few reports of associations between poor condition and decreased survival (e.g. Mathews *et al*. 2006) and correlational data that do not allow the discrimination between whether the impoverished health status was a cause or a consequence of disease (e.g. Hakkarainen *et al*. 2007). To our knowledge, this is the first study that evaluates longitudinally, and at the individual level, the condition-dependent risk of infection for a wildlife species.

The environment causes seasonal changes in the condition of voles (Beldomenico *et al*. in press), which in many cases result from the impact that voles have had on their own habitat, especially their food (Huitu *et al*. 2007; Beldomenico *et al*. in press). It has been suggested that the effect of the environment on immunocompetence is key to explaining the natural regulation of animal populations (Lochmiller 1996). Nutrient limitations can lead to reduced immune function (Gershoff *et al*. 1968; Jose & Good 1973; Gross & Newberne 1980; Lochmiller & Dabbert 1993). Furthermore, environmental stressors can negatively affect the immune system of rodents through sustained disruption of the neuroendocrine system (Geller & Christian 1982; McLean 1982; Stewart *et al*. 1988; Barnard *et al*. 1995, 1996). An impairment in fitness by a deficient diet and a suppressed immune system is reflected in the haemogram by the presence of anaemia and lymphopoenia, respectively (Feldman *et al*. 2000; Thrall 2004). Here, we have presented data indicating that poor condition increases the probability of infection: increased probabilities of developing monocytosis and higher increments in neutrophils in individuals with anaemia and lymphopoenia.

However, there were two exceptions to the pattern of lower condition being followed by an increased probability of a response suggestive of infection. First, monocytosis was least likely of all in lymphopoenic individuals at high densities (figure 1*b*). It has been shown previously that increases in monocyte levels in spring were the lowest for individuals with poor body condition, and at higher densities (Beldomenico *et al*. in press). This was interpreted as indicative of an impaired immune response, rather than an indicative of lower infection incidence. Similarly, the low likelihood of monocytosis in lymphopoenic individuals at high densities is most likely to be due to a hampered immune response.

Second, in spring 2006, normocytic females (not anaemic ones) were more likely to develop monocytosis. It has been argued previously (Beldomenico *et al*. in press), first, that in spring 2006 there seems to have been a metabolic demand that exceeded what could be supported by available resources and, second, that only females in good condition bred early (Beldomenico *et al*. in press). Hence, this difference between anaemic and normal females might plausibly be a consequence of greater exposure to infection in those in a condition good enough to start breeding, as the onset of breeding is associated with rises in indicators of infection (Beldomenico *et al*. in press).

Turning to the effect of infection on condition, high indices of infection (neutrophilia and monocytosis) were generally followed by a declining tendency in indicators of condition (RBCs and lymphocytes). However, falls in lymphocyte levels following neutrophilia were only evident in 2006, when the metabolic demands appeared not to be covered by the resources during neutrophil peaks (Beldomenico *et al*. in press) and when lymphopoenia was associated with poor survival (Beldomenico 2007). Moreover, in light individuals, individuals without monocytosis were more likely to show declines in RBC levels, particularly in spring. This might be related to a failure to mount an immune response by those in poor condition (Beldomenico *et al*. in press). On the other hand, spring was the only season when neutrophilia appeared to have a significant (negative) impact on RBCs, an effect that became more patent at higher densities.

Overall, then, our results suggest a vicious circle: poor condition predisposes to infection—infection predisposes to a decline in condition. They also therefore demonstrate that a careful interpretation of correlational evidence in wildlife disease studies is needed, and they highlight in particular the importance of longitudinal studies in determining temporal coherence between the putative cause and its alleged effect, vital to establish whether an association is causal or not. For example, Hakkarainen *et al*. (2007) recently reported an association of *Eimeria* spp. infection and lowered mother's and offspring's body conditions in bank voles, and interpreted this as supportive of the hypothesis that infections with *Eimeria* exert a significant loss of fitness. In fact, the association they observed might also have been a reflection of the vulnerability of those in poor condition (i.e. voles in poor condition were more prone to be infected and shed more *Eimeria* oocysts because their immune systems were weaker), or indeed that higher parasitism could have been both a cause and an effect of poor condition.

Our results, moreover, support Lochmiller's hypothesis that an underlying dynamic in condition (including immunocompetence) is key to explaining the natural regulation of animal populations (Lochmiller 1996; since poor condition predisposes an individual to infection) and the more general hypothesis that infectious disease may play a role in host dynamics (since infection also predisposes an individual to poor condition). But the vicious circle that links the two suggests that while pathogens overall may be more important in wildlife dynamics than has previously been appreciated, specific pathogens are likely to play their part as elements of an interactive web rather than independent entities.

Finally, this concept of vicious circles making pathogens the key determinants of host population dynamics seems likely to transcend the taxonomic group investigated here. For example, worldwide amphibian decline has been related to climate-linked epidemics (Pounds *et al*. 2006). While the prevalences to various pathogens were greater in declining amphibian populations than in non-declining ones (Di Rosa *et al*. 2007) (an indication that pathogens are affecting amphibian populations), it has been shown that global warming can degrade toads' condition (Reading 2007) and that frog declines were preceded by periods of increasing stress (Alford *et al*. 2007) (an indication that poor condition is implicated in population declines). Thus, an impoverished condition resulting from climate change may be triggering vicious circles that are decimating amphibian populations.

## Figures and Tables

**Table 1:** GLMM describing factors associated with the development of monocytosis in the four forthcoming weeks for field voles without monocytosis sampled from March/April to October in 2005 and 2006.

term	coefficients (log-odds)	s.e.	*p*-value	ΔAIC^a^
model=Monocytosis(yes/no)∼SEASON[sin]+SEASON[cos]+WGT+WGT^2^+DENSITY-0+SEX+YEAR+RBC+Lymphs+Lymphs^2^+R/N+DENSITY-0×L^2^+WGT×DENSITY-0+SEX×RBC+WGT×R/N+WGT^2^×R/N+SEASON[sin]×RBC+SEASON[cos]×DENSITY+SEASON[cos]×YEAR+YEAR×RBC+DENSITY×YEAR				
random effects=SITE×SESSION; VOLE_ID				
*N*=771				
intercept	0.692	2.388	0.7719	—
SEASON[sin]	2.000	0.523	0.0001	—
SEASON[cos]	0.815	0.590	0.1674	—
WGT	0.017	0.130	0.8958	—
WGT^2^	0.001	0.002	0.5676	—
DENSITY-0	−0.068	0.018	0.0002	—
SEX (male)	0.513	0.418	0.2197	—
YEAR (second)	−3.866	0.945	<0.0001	—
*RBC*	−0.009	0.060	0.8752	—
lymphocytes	0.0002	0.0001	0.0532	3.8
lymphocytes^2^	−5×10^−8^	2×10^−8^	0.0181	—
R/N	5.579	1.969	0.0046	—
SEASON[sin]×RBC	−0.203	0.060	0.0007	11.9
SEASON[cos]×DENSITY-0	−0.025	0.008	0.0022	9.6
SEASON[cos]×YEAR (second)	2.060	0.651	0.0016	10.9
WGT×DENSITY-0	0.001	5×10^−4^	0.0196	5.6
WGT×R/N	−0.426	0.148	0.0039	8.5
WGT^2^×R/N	0.008	0.003	0.0030	9.1
DENSITY-0×YEAR (second)	0.042	0.012	0.0007	12.5
DENSITY-0×lymphocytes^2^	4×10^−10^	1.5×10^−10^	0.0117	6.7
SEX (male)×*RBC*	−0.123	0.055	0.0270	5.0
YEAR×*RBC*	0.223	0.063	0.0004	13.4

a AIC value increment if the single term is dropped.

**Table 2:** LMM describing factors associated with changes in neutrophil levels in the four forthcoming weeks for field voles sampled from March to October in 2005 and 2006.

term	coefficients	s.e.	*p*-value	ΔAIC^a^
model=(Neutrophils sample 2−Neutrophils sample 1)∼SEASON[sin]+SEASON[cos]+WGT+WGT^2^+DENSITY-2+SEX+RBC+Lymphs+R/N+SEASON[sin]×WGT^2^+SEASON[sin]×SEX+WGT^2^×SEX+WGT×Lymphs+WGT^2^×Lymphs+DENSITY×R/N+SEX×R/N				
random effects=SITE×SESSION; VOLE_ID				
*N*=1047				
intercept	3000	1305	0.0217	—
SEASON[sin]	83	407	0.8391	—
SEASON[cos]	352	193	0.0752	—
WGT	−159	97	0.1016	—
WGT^2^	3.6	1.8	0.0472	—
DENSITY-2	−3.3	5.3	0.5381	—
SEX (male)	−148	142	0.2973	5.7
*RBC*	−66	23	0.0048	—
lymphocytes	−0.5	0.2	0.0401	—
R/N	448	192	0.0199	—
SEASON[sin]×WGT^2^	−0.8	0.4	0.0294	2.9
SEASON[sin]×SEX (male)	−343	153	0.0255	3.0
WGT^2^×SEX (male)	0.45	0.20	0.0235	3.3
WGT×lymphocytes	0.04	0.02	0.0437	2.1
WGT^2^×lymphocytes	−0.0008	0.0004	0.0174	3.7
DENSITY-2×R/N	−5.6	2.7	0.0389	2.3
SEX (male)×R/N	256	86	0.0030	7.2

a AIC value increment if the single term is dropped.

**Table 3:** LMMs describing factors associated with changes in RBC (first model) and lymphocyte (second model) levels in the four forthcoming weeks for field voles sampled from March to October in 2005 and 2006.

term	coefficients	s.e.	*p*-value	ΔAIC^a^
RBCs				
model=RBC difference∼SEASON[sin]+SEASON[cos]+WGT+WGT^2^+DENSITY-0+Nphilia+Mcytosis +SEASON[sin]×Mcytosis+SEASON[cos]×Nphilia+DENSITY-0×Nphilia+WGT×Mcytosis+WGT^2^×Mcytosis+DENSITY-0×Mcytosis				
random effects=SITE×SESSION; VOLE_ID				
*N*=923				
intercept	−6.70	3.19	0.0361	—
SEASON[sin]	2.10	0.72	0.0035	—
SEASON[cos]	−0.03	0.70	0.9649	—
WGT	0.30	0.19	0.1141	—
WGT^2^	−0.005	0.003	0.0967	—
DENSITY-0	0.04	0.02	0.0485	—
neutrophilia (yes)	1.70	1.20	0.1564	—
monocytosis (yes)	11.87	4.25	0.0053	—
SEASON[sin]×Monocytosis	−1.45	0.70	0.0376	2.3
SEASON[cos]×Neutrophilia	−1.78	0.69	0.0101	4.6
DENSITY—0×Neutrophilia	−0.03	0.01	0.0322	2.5
WGT×Monocytosis	−0.68	0.27	0.0119	4.4
WGT^2^×Monocytosis	0.01	0.004	0.0170	3.8
DENSITY-0×Monocytosis	−0.03	0.01	0.0304	2.7
lymphocytes				
model=Lymph difference∼SEASON[sin]+SEASON[cos]+WGT+DENSITY-6+YEAR+Nphilia+Mcytosis +SEASON[cos]×WGT+SEASON[cos]×YEAR+WGT×DENSITY-6+WGT×YEAR+YEAR×Nphilia				
random effects=SITE×SESSION; VOLE_ID				
*N*=970				
intercept	6929	2036	0.0007	—
SEASON[sin]	1065	315	0.0007	10.1
SEASON[cos]	3674	954	0.0001	—
WGT	−159	74	0.0307	—
DENSITY-6	−84	25	0.0009	—
YEAR (first)	−5354	1555	0.0006	—
neutrophilia (yes)	−1621	349	<0.0001	—
monocytosis (yes)	−1900	260	<0.0001	—
SEASON[cos]×WGT	−103	34	0.0027	7.6
SEASON[cos]×YEAR (first)	−1153	546	0.0348	2.9
WGT×DENSITY-6	2.3	0.9	0.0125	4.9
WGT×YEAR (first)	118	55	0.0319	3.2
YEAR (first)×Neutrophilia	1469	574	0.0107	4.7

a AIC value increment if the single term is dropped.
